# Chemotherapy for gliomas in mainland China: An overview

**DOI:** 10.3892/ol.2013.1264

**Published:** 2013-03-19

**Authors:** KE SAI, QUN-YING YANG, DONG SHEN, ZHONG-PING CHEN

**Affiliations:** Department of Neurosurgery/Neuro-Oncology, Sun Yat-Sen University Cancer Center, State Key Laboratory of Oncology in South China, Guangzhou 510060, P.R. China

**Keywords:** glioma, chemotherapy, mainland China

## Abstract

Chemotherapy is currently the standard treatment modality for malignant gliomas. Many patients with gliomas are treated in mainland China every year. The history and development of chemotherapy for glioma, however, are not well documented. In this study, an extensive literature search of Pubmed and major Chinese electronic databases was performed to identify clinical studies. A total of 210 publications were identified, with a total of 10,105 patients. Among these studies, 76.2% were retrospective and 23.8% were prospective. Chemotherapy was found to have been administered by the Department of Neurosurgery in 143 studies (68.1%). Oral or intravenous administration was found in 55.7% of studies, followed by intra-arterial (26.7%) and interstitial (15.7%) chemotherapy. Nitrosoureas were the most frequently used chemotherapeutic agents, as found in 133 studies (63.3%). Since 2003, 56 studies on temozolomide (TMZ) have been published. Studies on chemotherapy for gliomas began in the 1970s in mainland China but well-designed randomized controlled trials (RCTs) are rare. Much effort and collaboration should be made to carry out high-quality multicenter RCTs on chemotherapy for gliomas.

## Introduction

Gliomas are the most frequent primary intracranial tumors in adults, accounting for 80% of all primary malignant tumors in the central nervous system (CNS) (1). The management of gliomas is sometimes challenging due to the unconstrained growth and infiltrative nature of glioma cells. Traditionally, debulking surgery and post-operative radiation are the mainstay in the treatment of gliomas but the prognosis is far from satisfactory. CNS cancers, with a majority of gliomas, remain the second most common lethal cancer in males younger than 40 years (2). Historically, the efficacy of chemotherapy for gliomas is controversial due to the blockade of chemotherapeutic agents by the blood-brain barrier (BBB) and the insensitivity of gliomas to chemotherapy (3). However, the role of chemotherapy for gliomas has been established with efforts in basic research and clinical studies. In 1994, Cairncross *et al* demonstrated that oligodendrial gliomas are sensitive to a chemotherapeutical regimen containing procarbazine, lomustine and vincristine (4). In a phase II trial, Yung *et al* showed that chemotherapy with temozolomide (TMZ) improved the prognosis of patients with anaplastic gliomas (5). In 2005, the cornerstone prospective randomized clinical trial performed by Stupp *et al* revealed that TMZ combined with radiation significantly improves the prognosis of newly diagnosed glioblastoma multiforme (GBM), with a 5-year overall survival (OS) of 9.8%, compared with that of 1.9% for radiotherapy alone (6). Chemotherapy has now become the standard of care for malignant gliomas. In mainland China, numerous patients with gliomas are treated every year and increasing attention has been paid to chemotherapy. However, the history and development of chemotherapy for gliomas in mainland China are not well documented. In this study, a thorough literature search was performed and a review of the field of glioma chemotherapy in mainland China was conducted.

## Materials and methods

### Literature search

In August 2011, an extensive literature search was performed to identify clinical studies reporting outcomes of glioma patients treated with chemotherapy in mainland China. The electronic databases of Pubmed, China Knowledge Resource Integrated Database, Chinese Medical Association Digital Periodicals and VIP Database for Chinese Technical Periodicals were searched. Keywords searched included ‘glioma’, ‘glial tumor’, ‘glioblastoma’, ‘astrocytoma’, ‘oligodendroglioma’, ‘oligodendroastrocytoma’, ‘chemotherapy’, ‘drug therapy’ and ‘drug treatment’.

### Selection criteria

There were no language restrictions for the searched articles. Titles and abstracts were first examined to exclude irrelevant diseases and treatment, and duplicates were excluded. Studies selected were in accordance with the following criteria: i) A clinical study had been conducted on chemotherapy for intracranial gliomas in mainland China; ii) The number of patients was ≥5; iii) >70% of patients were adults (≥18 years); iv) Patients with glioma comprised ≥70% of all cases.

### Data extraction and analysis

Information of publications, patient and chemotherapy information was extracted. Collected data were analyzed and reviewed.

## Results

### Publication selection

**Figure 1:**
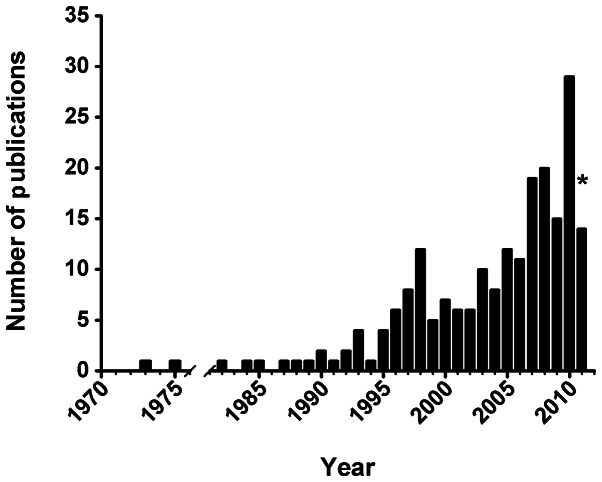
The number of studies on chemotherapy for gliomas published each year from 1970–2011. ^*^Up to August 2011.

**Figure 2:**
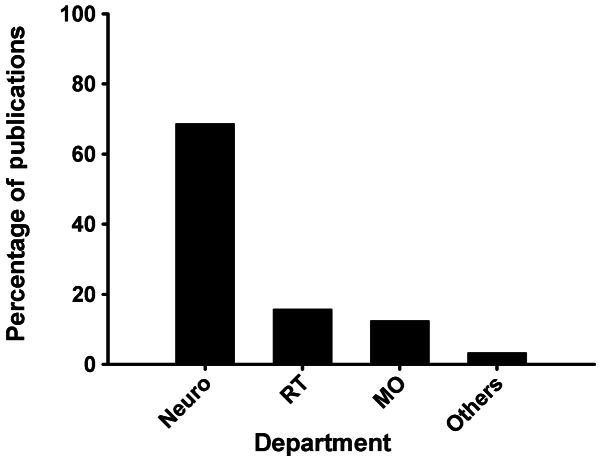
Departments where chemotherapy was administered. Neuro, neurosurgery; RT, radiotherapy; MO, medical oncology.

A total of 333 potentially eligible publications were found using the search strategy and by screening titles and abstracts. A total of 210 articles were identified to be in line with the selection criteria, of which 160 (76.2%) were retrospective and 50 (23.8%) were prospective. An increasing number of publications have been published over time, with only 2 studies published before 1980 but 29 in 2010 (Fig. 1). Of the 210 studies, 144 (68.6%) were performed in the Department of Neurosurgery, 33 (15.7%) in the Department of Radiotherapy and 26 in the Department of Medical Oncology (Fig. 2).

### Patient data

**Figure 3:**
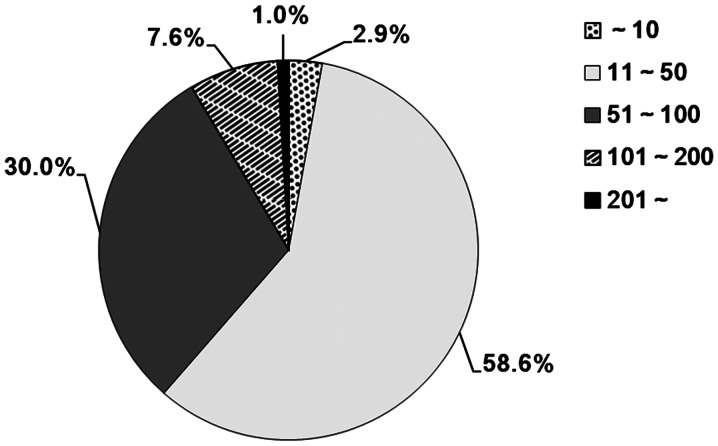
The number of patients enrolled in studies.

In all, 10,105 patients with glioma were enrolled in the 210 studies. The mean age of patients was 21–56 years and the male/female ratio was 1.5:1. Of the 210 studies, 192 (91.4%) enrolled fewer than 100 patients and only 18 (8.6%) had >100 cases in each study (Fig. 3).

### Chemotherapy information

**Figure 4:**
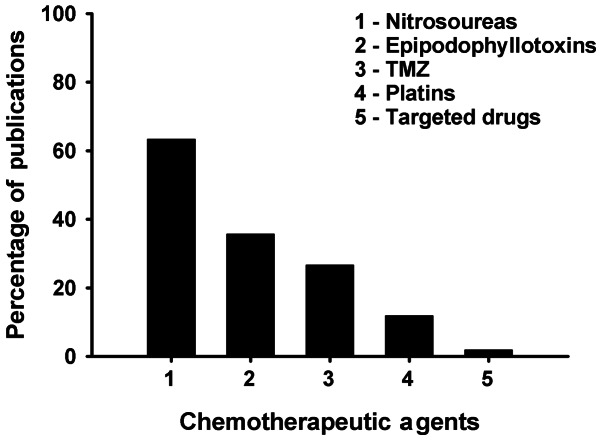
Chemotherapeutic agents used in studies. TMZ, temozolomide.

**Figure 5:**
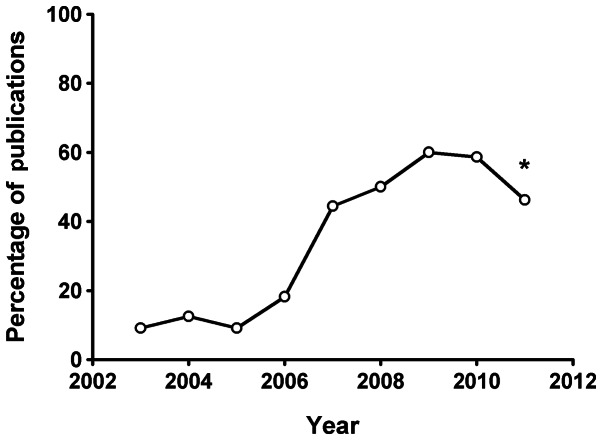
Trend in the publications of studies on temozolomide (TMZ) in the treatment of gliomas. ^*^Up to August 2011.

**Figure 6:**
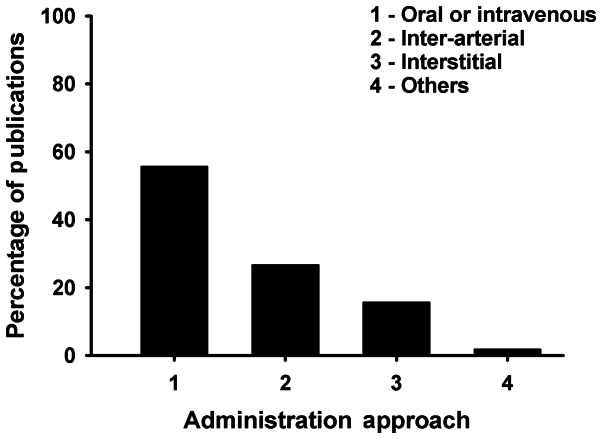
Approaches employed to administer chemotherapeutic agents in studies.

**Figure 7:**
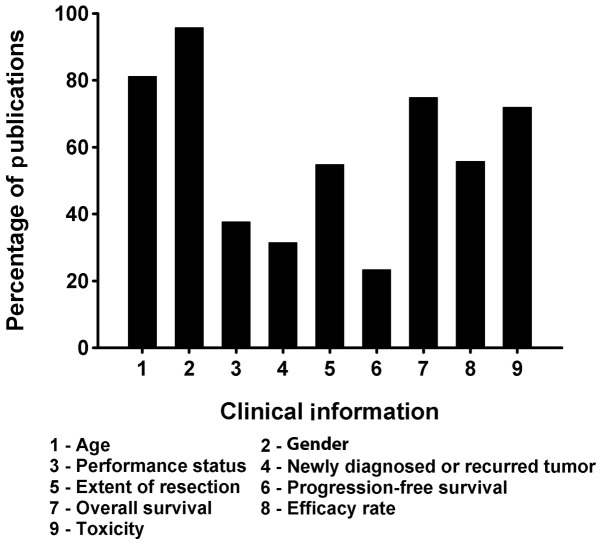
Clinical information reported in studies.

Nitrosourea drugs including nimustine (ACNU), carmustine (BCNU), lomustine (CCNU) and semustine (MeCCNU) were the most frequently used chemotherapeutic agents and were found in 133 (63.3%) studies. The epipodophyllotoxins were used in 75 (35.7%) studies, TMZ in 56 (26.7%), platins in 25 (11.9%) and targeted agents in 4 (1.9%) studies, respectively (Fig. 4). Nitrosourea-containing regimens decreased from 100% of studies before 1990 to 59.4% of publications since 2000. Since 2003, TMZ has been used to treat gliomas in mainland China and the number of publications regarding TMZ for gliomas has gradually increased (Fig. 5). The majority of chemotherapy was administered orally or intravenously, which was found in 117 (55.7%) studies. Intra-arterial and interstitial administration were found in 56 (26.7%) and 33 (15.7%) studies, respectively (Fig. 6). Important characteristics of patients were reported in most but not all studies. Of the 210 studies, 11 studies contained gliomas of only one pathological grade and 190 studies had gliomas of at least two pathological grades. Among the 210 studies, patient age was reported in 170 (81.1%) studies, gender in 201 (95.7%), Karnofsky performance score (KPS) in 79 (37.6%), newly diagnosed or recurring tumors in 66 (31.4%), extent of tumor resection in 115 (54.8%), progression-free survival (PFS) in 49 (23.3%), overall survival (OS) in 157 (74.8%), efficacy rate in 117 (55.7%) and toxicity in 151 (71.9%) studies, respectively (Fig. 7).

## Discussion

Although chemotherapy has been employed as an adjuvant treatment for glioma for more than three decades, its efficacy as chemotherapy for glioma has been controversial. In 2002, Stewart performed a systematic review of 12 randomized trials and demonstrated that chemotherapy significantly prolonged the survival of adult patients with high-grade gliomas (7). In 2005, a randomized clinical trial demonstrated the efficacy of TMZ, making TMZ the standard of care for GBM (8). In addition, the success of biodegradable BCNU wafers (Gliadel) and bevacizumab (Avastin) strengthened the role of chemotherapy in the management of gliomas (9,10). Increasing attention has been paid to studies on glioma chemotherapy worldwide. There have been no studies to date describing the history and development of chemotherapy for gliomas in mainland China. In this study, the major electronic medical databases were searched for studies on glioma chemotherapy in mainland China and an analysis was conducted to provide an overview.

Studies on chemotherapy for gliomas began at the beginning of the 1970s in mainland China. In 1973, a group from Tianjin Medical School introduced their experience of using BCNU to treat brain tumors (11). In 1975, a study published by neurosurgeons from Suzhou Medical School presented preliminary results of the management of gliomas with CCNU (12). These two retrospective studies demonstrated the potential antitumor activity of nitrosoureas in Chinese glioma patients and described the most common side-effects, including gastrointestinal adverse reactions and myelosuppression. In the following years, publications of glioma chemotherapy in mainland China increased significantly. Only eight studies were published during the 20 years from 1970–1990 while 29 studies were identified in 2010 alone.

Similar to the situation in most Asian countries, chemotherapy in mainland China was mainly administered by neurosurgeons. Among the 210 studies identified, 144 (68.6%) were carried out in the Department of Neurosurgery, followed by 33 (15.7%) in the Department of Radiation Oncology and 26 (12.4%) in the Department of Medical Oncology. One of the reasons for this may be that neurosurgeons are capable of administering chemotherapeutic agents through routes other than oral and intravenous, such as arterial and interstitial delivery. Another reason may be that neurological complications during chemotherapy and disease progression may be managed by neurosurgeons with more confidence and efficiency. With the development of neuro-oncology in mainland China in the past 10 years, physicians with expertise of oncology have been trained and recruited by the Department of Neurosurgery to administer chemotherapy to glioma patients. The increasing number of neuro-oncologists in mainland China will provide glioma patients with better and more specialized care and focus on both clinical and basic research in the field of neuro-oncology.

Nitrosoureas were the most common agents used as chemotherapy for Chinese glioma patients. The earliest two studies identified explored the efficacy of nitrosoureas (11,12). Nitrosoureas were predominantly employed in the majority of studies, accounting for 63.3% of the 210 studies. However, due to the toxicity and the chemoresistance of glioma cells, the number of reports of nitrosourea-containing regimens has declined from 100% of studies published before 1990 to 59.4% of publications after 2000. Other chemotherapeutic agents have been adopted to treat Chinese glioma patients. Of the 210 studies, epipodophyllotoxins were used in 75 (35.7%) and platins in 25 (11.9%). The earliest report focusing on TMZ, a novel oral alkylating agent with low toxicity for the management of Chinese patients with gliomas was published in 2003. In the study, the authors retrospectively investigated the anti-tumor activity of TMZ in 17 patients with malignant gliomas. An objective response rate of 47.1% and a 6-month survival rate of 58.8% were observed (13). Once the efficacy of TMZ was confirmed by a large well-designed clinical trial by Stupp *et al*, the publications of chemotherapy with TMZ in Chinese glioma patients significantly increased (6). In 2003, 9.1% of studies explored chemotherapy with TMZ while 58.6% of publications in 2010 used TMZ-containing regimens.

Multiple approaches have been investigated to deliver chemotherapeutic agents for gliomas in mainland China. Among the 210 studies examined, oral or intravenous administration was found in 55.7% of studies, intra-arterial administration in 26.7% and interstitial administration in 15.7% of studies. There are pharmacological rationales for intra-arterial administration. When a drug is delivered intraarterially, it results in an augmentation of the local peak plasma concentration and the area under the curve (AUC) of the drug, compared with the conventional intravenous dose. Mathematical modeling predicted up to a five-fold increase in drug delivery by intra-arterial administration as defined by the concentration-time integral (14). A study using positron emission tomography and ^11^C-labeled BCNU demonstrated that superselective intra-arterial infusion in patients with recurrent gliomas resulted in an ∼50-fold increase in drug administration compared with intravenous delivery (15). Although retrospective intra-arterial studies reported some positive results in mainland China, no survival benefit of intra-arterial infusion for glioma patients has been proven in phase III trials overseas (16). Thus, intra-arterial chemotherapy for patients with glioma is diminishing in mainland China.

Interstitial chemotherapy is also an appealing method to administer chemotherapeutic drugs because local delivery of drugs in the tumor bed potentially overcomes systemic toxicities and limitations in traversing the blood-brain barrier by systemic agents. Until now, Gliadel wafers have been the most successful commercially available interstitial chemotherapy drug for gliomas. A large phase III clinical trial demonstrated that this BCNU-loaded polymer prolonged the median survival of patients with primary malignant glioma from 11.6 to 13.9 months compared with the control group without significant toxicity (17). Based on its safety and efficacy, Gliadel wafers are recommended for newly diagnosed and recurrent GBM by National Comprehensive Cancer Network (NCCN) guidelines. In mainland China, the following interstitial drug delivery methods in glioma patients were investigated: i) Administration of chemotherapeutic agents through stereotaxic surgery (18); ii) Implantation of materials containing drugs in the tumor bed during neurosurgery (19); iii) Infusion of chemotherapeutic agents to the post-operative cavity through a subcutaneous reservoir (20). Potential efficacy was observed in the interstitial chemotherapy studies. The small sample size and the retrospective nature of these studies, however, makes prospective trials necessary to confirm the results. Recently, unpublished phase I trial data revealed that biodegradable BCNU-loaded polymers, developed by Chinese investigators, with a higher dosage and more stable release of drug compared with Gliadel wafers, are well-tolerated in patients with recurrent malignant gliomas. A large randomized trial has been launched to evaluate the efficacy of this interstitial chemotherapy.

There are challenges in chemotherapy studies for glioma patients in mainland China. Firstly, the majority of the identified studies were retrospective with a small sample size. Of the 210 studies, only 50 (23.8%) were prospective. In addition, despite a total of 10,105 cases enrolled, 91.4% of all 210 studies enrolled fewer than 100 patients and only two studies had a sample size of >200 patients. The retrospective nature and small sample size will result in bias and weaken the scientific strength of the studies. Secondly, improvement in the reporting of studies is required. A proper report of the methodology and detailed results of a study are necessary in order to understand the significance of the study and to compare it with others. In the studies identified, certain important information was missing. For example, performance status and extent of tumor resection were only reported in 37.6 and 54.8% of all 210 studies, respectively. These are well-known prognostic factors for glioma patients (21). If this information is missing, the quality and reliability of a study may potentially be impaired. Thirdly, more effort is required for studies on targeted therapy in glioma patients in mainland China. With advances in molecular biology, disruption of signaling pathways has been revealed to be responsible for the development, progression and treatment resistance of gliomas. Strategies specifically targeting abnormalities in molecular pathways theoretically have better efficacy and safety profiles than systemic cytotoxic chemotherapy (22). The success of bevacizumab, a monoclonal antibody targeting angiogenic pathways, has created an evolving therapeutic landscape for gliomas. Many international clinical trials on the efficacy of small molecule drugs are ongoing. Studies on targeted therapy for glioma patients in mainland China has just begun. Only 1.9% of the studies identified according to our selection criteria involved inhibitors targeting epidermal growth factor (EGF) or other potential angiogenic pathways.

In this study, an overview of chemotherapy for glioma in mainland China is presented. The role of chemotherapy in the management of gliomas is well-recognized. The investigation into the efficacy of chemotherapy in glioma patients began in the 1970s in mainland China and multiple disciplines participate. Different chemotherapeutic agents and various methods of drug delivery have been tested. The rarity of randomized controlled trials (RCTs) with high quality results is a major limitation. Much effort and collaboration should, therefore, be made to conduct well-designed multicenter RCTs on chemotherapy, with the aim of improving the prognosis of patients with glioma.

## References

[1] Schwartzbaum JA, Fisher JL, Aldape KD, Wrensch M (2006). Epidemiology and molecular pathology of glioma. Nat Clin Pract Neurol.

[2] Siegel R, Naishadham D, Jemal A (2012). Cancer statistics, 2012. CA Cancer J Clin.

[3] Minniti G, Muni R, Lanzetta G, Marchetti P, Enrici RM (2009). Chemotherapy for glioblastoma: current treatment and future perspectives for cytotoxic and targeted agents. Anticancer Res.

[4] Cairncross G, Macdonald D, Ludwin S (1994). Chemotherapy for anaplastic oligodendroglioma. National Cancer Institute of Canada Clinical Trials. Group J Clin Oncol.

[5] Yung WK, Prados MD, Yaya-Tur R (1999). Multicenter phase II trial of temozolomide in patients with anaplastic astrocytoma or anaplastic oligoastrocytoma at first relapse. Temodal Brain Tumor. Group J Clin Oncol.

[6] Stupp R, Hegi ME, Mason WP (2009). Effects of radiotherapy with concomitant and adjuvant temozolomide versus radiotherapy alone on survival in glioblastoma in a randomised phase III study: 5-year analysis of the EORTC-NCIC trial. Lancet Oncol.

[7] Stewart LA (2002). Chemotherapy in adult high-grade glioma: a systematic review and meta-analysis of individual patient data from 12 randomised trials. Lancet.

[8] Stupp R, Mason WP, van den Bent MJ (2005). Radiotherapy plus concomitant and adjuvant temozolomide for glioblastoma. N Engl J Med.

[9] Hart MG, Grant R, Garside R, Rogers G, Somerville M, Stein K (2011). Chemotherapy wafers for high grade glioma. Cochrane Database Syst Rev CD.

[10] Friedman HS, Prados MD, Wen PY (2009). Bevacizumab alone and in combination with irinotecan in recurrent glioblastoma. J Clin Oncol.

[11] Tianjin Medical School Affliated Hospital (1973). Preliminary study on BCNU in the treatment of intracranial tumors: report of 45 cases. Tianjin Med J.

[12] The First Affliated Hospital to Suzhou Medical School (1975). Preliminary observation of treating gliomas in central nervous system with CCNU. Chin J Nerv Ment Dis.

[13] Zeng X, Yang S (2003). Clinical observation in chemotherapy with temozolomide alone in postoperative malignant primary cerebral glioma. Mod J Neurol Neurosurg.

[14] Newton HB (2005). Intra-arterial chemotherapy of primary brain tumors. Curr Treat Options Oncol.

[15] Tyler JL, Yamamoto YL, Diksic M (1986). Pharmacokinetics of superselective intra-arterial and intravenous [11C]BCNU evaluated by PET. J Nucl Med.

[16] Shapiro WR, Green SB, Burger PC (1992). A randomized comparison of intra-arterial versus intravenous BCNU, with or without intravenous 5-fluorouracil, for newly diagnosed patients with malignant glioma. J Neurosurg.

[17] Westphal M, Hilt DC, Bortey E (2003). A phase 3 trial of local chemotherapy with biodegradable carmustine (BCNU) wafers (Gliadel wafers) in patients with primary malignant glioma. Neuro Oncol.

[18] Lin Y, Chen W, Zhuge Q (1996). Combined CT-guided stereo-tactic interstitial radiotherapy of 32P and MTX chemotherapy for treatment of deep brain gliomas. Chin J Neurosurg.

[19] Qi S, Qiu B (2004). Treatment of recurrent malignant brain gliomas by surgical excision combined with biodegradable polymers of interstitial chemotherapy. Zhonghua Zhong Liu Za Zhi.

[20] Li A, Zhang X, Yi Y (1999). Interstitial chemotherapy for malignant chemotherapy. Chin J Clin Neurosurg.

[21] Buckner JC (2003). Factors influencing survival in high-grade gliomas. Semin Oncol.

[22] Van Meir EG, Hadjipanayis CG, Norden AD, Shu HK, Wen PY, Olson JJ (2010). Exciting new advances in neuro-oncology: the avenue to a cure for malignant glioma. CA Cancer J Clin.

